# Imprecision in Precision Medicine: Differential Response of a Disease-Linked GluN2A Mutant to NMDA Channel Blockers

**DOI:** 10.3389/fphar.2021.773455

**Published:** 2021-10-28

**Authors:** Jenna R. Gale, Gabrielle J. Kosobucki, Karen A. Hartnett-Scott, Elias Aizenman

**Affiliations:** Department of Neurobiology and Pittsburgh Institute for Neurodegenerative Diseases, University of Pittsburgh School of Medicine, Pittsburgh, PA, United States

**Keywords:** GRIN2A gene, GluN2A subunit, memantine, ketamine, precision medicine, N-methyl-d-aspartate receptor, channelopathy

## Abstract

Mutations in N-methyl-d-aspartate receptors (NMDAR) subunits have been implicated in a growing number of human neurodevelopmental disorders. Previously, a *de novo* mutation in *GRIN2A*, encoding the GluN2A subunit, was identified in a patient with severe epilepsy and developmental delay. This missense mutation, which leads to GluN2A-P552R, produces significant dendrotoxicity in transfected rodent cortical neurons, as evidenced by pronounced dendritic blebbing. This injurious process can be prevented by treatment with the NMDA antagonist memantine. Given the increasing use of FDA approved NMDA antagonists to treat patients with *GRIN* mutations, who may have seizures refractory to traditional anti-epileptic drugs, we investigated whether additional NMDA antagonists were effective in attenuating neurotoxicity associated with GluN2A-P552R expression. Intriguingly, we found that while treatment with memantine can effectively block GluN2A-P552R-mediated dendrotoxicity, treatment with ketamine does not, despite the fact that both drugs work as open NMDAR channel blockers. Interestingly, we found that neurons expressing GluN2A-P552R were more vulnerable to an excitotoxic insult—an effect that, in this case, could be equally rescued by both memantine and ketamine. These findings suggest that GluN2A-P552R induced dendrotoxicity and increased vulnerability to excitotoxic stress are mediated through two distinct mechanisms. The differences between memantine and ketamine in halting GluN2A-P552R dendrotoxicity could not be explained by NMDA antagonist induced changes in MAP or Src kinase activation, previously shown to participate in NMDA-induced excitotoxicity. Our findings strongly suggest that not all NMDA antagonists may be of equal clinical utility in treating *GRIN2A*-mediated neurological disorders, despite a shared mechanism of action.

## Introduction

N-methyl-d-aspartate receptors (NMDARs) are ligand-gated, ionotropic glutamate receptors that are widely expressed in the brain, where they play key roles in neuronal developmental, synaptic plasticity, and survival. NMDARs are heterotetramers composed of three main subtypes: GluN1, which are obligatory, GluN2 of which there are four subunit types (A-D), and GluN3 subunits of which there are two subunit types (A-B) (Traynelis et al., 2010; Paoletti et al., 2013). The majority of NMDARs express two glycine-binding GluN1 subunits and two glutamate-binding GluN2 subunits (Köhr, 2006). The GluN2 subtype dictates many of the NMDAR’s characteristics such as its biophysical, pharmacological, and signaling properties, as well as its spatiotemporal pattern of expression (Monyer et al., 1992; Watanabe et al., 1994; Paoletti et al., 2013; Vieira et al., 2020).

Mutations in NMDAR subunits have been increasingly implicated in neurological and neurodevelopmental diseases, including intellectual disability, autism spectrum disorders, developmental delay, and epilepsy (Myers et al., 2019). The identification of *GRIN* variants in pediatric patients is significant, as individuals *GRIN* mutations and epilepsy refractory to standard anti-convulsants have been successfully treated with the FDA-approved NMDAR antagonists memantine, ketamine, and dextromethorphan (Pierson et al., 2014; Li et al., 2016; Amador et al., 2020; Xu et al., 2021). Although mutations in four genes encoding NMDAR subunits (*GRIN1, GRIN2A, GRIN2B, and GRIN2D*) have been linked to human disease (Carvill et al., 2013; Li et al., 2016; Liu et al., 2017; Li et al., 2019; Bahry et al., 2021; Xu et al., 2021), mutations in *GRIN2A* account for the majority of disease-linked variants (46%) (Myers et al., 2019; Strehlow et al., 2019). Pathogenic variants cluster in the highly conserved agonist binding domains as well as transmembrane and linker domains, which are highly intolerant to genetic variation (Swanger et al., 2016; Ogden et al., 2017; Strehlow et al., 2019).

Recently, a *de novo* missense mutation in the pre-M1 helix region—the linker between the agonist binding domain and the first transmembrane domain—was identified in a patient with profound intellectual disability, developmental delay, and epilepsy (de Ligt et al., 2012). This mutation, which results in a substitution of arginine for proline at amino acid 552 (P552R), was further characterized as leading to neurotoxicity when expressed in cultured rat cortical neurons, as evidenced by pronounced dendritic swelling (Ogden et al., 2017). This neurotoxicity could be rescued by treatment with memantine. Given the superiority of ketamine as compared to memantine in treating some *GRIN* mutations (Li et al., 2016), and its widespread use in treating status epilepticus in adult and pediatric populations (Mewasingh et al., 2003; Ilvento et al., 2015; Zeiler, 2015), we sought to determine whether ketamine treatment was equally effective as memantine in abrogating GluN2A-P552R induced neurotoxicity *in vitro*. Surprisingly, our data indicate that the P552R mutation exerts neurotoxicity through two separate mechanisms, dendritic blebbing and increased susceptibility to excitotoxic injury—both responsive to treatment with memantine but only the latter responsive to treatment with ketamine, despite a shared mechanism of action between these two drugs.

## Methods

### Materials

Primary antibodies used: rabbit anti-p38 MAPK (Cell Signaling, 9212S, 1:1,000), mouse anti-phospho-p38 MAPK (Cell Signaling, 9216S, 1:1,000), mouse anti-ERK (pan-ERK) (BD Transduction Lab, 610,123, 1:2000), rabbit anti-phospho-p44/42 MAPK (ERK 1/2) (Cell Signaling, 910, 1:1,000), mouse anti-JNK (Santa Cruz, sc-7345, 1:1,000), rabbit anti-pSAPK/JNK (Cell Signaling, 4668S, 1:1,000), rabbit anti-Src (Cell Signaling, 2108S, 1:1,000), rabbit anti-phospho-Src (Tyr416) (Cell Signaling, 2101S, 1:1,000), and mouse anti-β-actin (Sigma, A5441, 1:10,000). Mouse or rabbit secondary antibodies used: Li-cor IRDye 700 CW and Licor IRDye 800CW (LI-COR Biosciences). Chemicals were obtained from Sigma-Aldrich unless otherwise specified.

### Neuronal Cultures

All procedures involving the use of animals were reviewed and approved by the University of Pittsburgh IACUC. Primary cortical cultures were prepared from embryonic day 16–17 Sprague-Dawley rats as previously described (Hartnett et al., 1997). Briefly, pregnant rats (Charles River Laboratories) were sacrificed via CO_2_ inhalation. Embryonic cortices were dissociated with trypsin and cells were plated on 12-mm, poly-L-ornithine coated glass coverslips in six-well plates at a density of 670,000 cells per well. On day 14 *in vitro* (DIV 14) cytosine arabinoside (1–2 µM) was used to inhibit nonneuronal cell proliferation. Cultures were used at DIV 18–25.

### Transfections

Transfections were performed using Lipofectamine 2000 (Thermo Fisher). Neurons were transfected with the following plasmid mixtures (total of 1.5 µg DNA/0.5 ml): for confocal imaging, cells were transfected with a GFP-expressing plasmid (pEGFP-N1; BD Biosciences), pCI-neo vector, human wild-type GluN2A, or human GluN2A-P552R (gifts from Drs. Hongjie Yuan and Stephen Traynelis). Plasmid mixtures (per well) contained either 0.9 µg pEGFP-N1 + 0.6 µg pCI-neo vector, 0.9 µg pEGFP-N1 + 0.3 µg pCI-neo empy vector + 0.3 µg GluN2A plasmid, or 0.9 µg pEGFP-N1 + 0.3 µg pCI-neo empy vector + 0.3 µg GluN2A-P552R plasmid (Ogden et al., 2017). For luciferase viability experiments, cells were transfected with a firefly luciferase-expressing plasmid (pUHC13-3 Luciferase, gift of Dr. H. Buchard) instead of one expressing GFP. Plasmid mixtures (per well) contained either 0.375 µg pUHC13-3 Luciferase + 1.125 µg pCI-neo vector plasmid, 0.375 µg pUHC13-3 Luciferase + 0.525 µg pCI-neo vector + 0.6 µg GluN2A plasmid, or 0.375 µg pUHC13-3 Luciferase + 0.525 µg pCI-neo vector plasmid + 0.6 µg GluN2A-P552R plasmid.

### Confocal Imaging and Bleb Analysis

To analyze the effect of the GluN2A-P552R mutation on dendrite morphology, cells were imaged 24 h after transfection. All treatments were added to the cell media at the time of transfection. Images were obtained on a Nikon A1+ confocal microscope using a ×20 water immersion objective. Three separate culture dates were used per experiment, and three coverslips were transfected with each plasmid mixture per condition. Each coverslip was divided into four quadrants, and one field of view was randomly selected from each quadrant resulting in four images per coverslip. Bleb counts per field were added to determine a bleb count per coverslip (blebs/CS). For these experiments, *n* refers to the number of coverslips. Laser power was sometimes adjusted between coverslips due to differences in transfection efficiency of the GFP-expressing plasmid. Nikon Instruments Software Basic Research (NIS-Elements BR) was used for non-biased analysis of dendritic blebs. All images were subjected to intensity thresholding before analysis. The object count feature was used to quantify the number of blebs in each image field. Area was restricted to 0–5 μm^2^ and circularity was set to 0.5–1.0. The smooth, clean, and separate features were used to reduce background noise and settings were consistent between control, ketamine, and MK-801 groups. Settings had to be adjusted once due to the installation of a new laser in our system.

### Lactate Dehydrogenase Assays

Cortical neurons were treated with 10 µM glycine, 10 µM glycine + 30 µM NMDA, or 10 µM glycine + 30 µM NMDA with either 50 µM memantine or 10 µM ketamine for 30 min in HEPES-buffered minimal essential media with 0.01% BSA (MHB). After 30 min, cells were washed with fresh MHB and then incubated with MHB or MHB containing memantine or ketamine. Twenty-four hs following glycine + NMDA treatment, medium was collected for lactate dehydrogenase (LDH) assays. Toxicity is represented by increased OD490 values. Three experiments from separate culture dates were performed, each in quadruplicate.

### Luciferase Viability Assays

Cortical cultures were transfected with plasmid mixtures containing a luciferase-expressing. Twenty-four hs following transfection, cells were either left untreated, treated with 45 µM DL-*threo*-β-Benzyloxyaspartate (TBOA) (Tocris Bioscience), or co-treated with 45 µM TBOA and either 50 µM memantine or 10 µM ketamine. TBOA is a glutamate uptake inhibitor that induces an excitotoxic injury *in vitro* (Bonde et al., 2003). Twenty-four hs after drug treatment, firefly luciferase expression was measured using the SteadyLite Plus Luminescence Gene Reporter Assay System (PerkinElmer) (Aras et al., 2008). Results for TBOA-treated groups were normalized to the luminescence values (counts per second) of their respective untreated groups. This assay was performed a minimum of four times with neurons from separate culture dates.

### Immunoblotting

Cortical cultures were left untreated, treated with 50 µM memantine, or treated with 10 µM ketamine for 45 min. Control, memantine treated-, and ketamine treated neurons were collected from the same 6 well plate for each experiment. Following treatment, neurons were rinsed twice with ice-cold PBS, exposed to cell lysis buffer (Invitrogen) supplemented with protease inhibitor cocktail (Roche Diagnostics) and phenylmethlysulfonyl fluoride, and scraped off dishes. Debris was pelleted by centrifugation at 14,000 g for 10 min. The remaining lysates were stored at −80°C. Protein concentration of lysates was determined (Pierce BCA Protein Assay Kit; Thermo Fisher). Samples were prepared by incubating 30 µg of protein with a reducing sample buffer at 100°C for 5 minutes. Samples were loaded onto a 7.5% SDS-PAGE gel and proteins were separated using the Mini Protean 3 System (Biorad). Gels were transferred onto 0.2 µm nitrocellulose membranes and blocked at room temperature for 1 h with 1% BSA in PBS containing 0.05% Tween 20 (PBST). Membranes were incubated overnight at 4°C in primary antibody. After washing 3x in PBST, membranes were probed with Li-Cor IRDye-conjugated secondary antibodies labeled with IRDyes 700CW (685 nm) and 800CW (780 nm), at 1:10,000 for 1 h at room temperature. Fluorescent signals were acquired (Odyssey Infrared Imaging System; LI-COR) and quantified using Fiji software.

### Statistical Analysis

Data are presented as means ± SEM. All statistical analyses were performed using GraphPad Prism 9 (GraphPad). Prior to analyses, data were tested for normality using a Shapiro Wilk test. For comparison of two sample means, a two-tailed *t*-test was used. For comparison of more than two sample means, a one-way analysis of variance (ANOVA) with Tukey’s test for multiple comparisons was used. For LDH assays, a two-way ANOVA with Sidak’s multiple comparison test was used to compare the effect of drug treatments on viability between control and treatment groups.

## Results

A previous characterization of the GluN2A-P552R mutation found that the mutant NMDAR subunit exerted profound dendrotoxicity when expressed in primary neurons, as evidenced by dendritic swelling (blebbing) (Ogden et al., 2017). GluN2A-P552R mediated dendritic blebbing could be rescued by treating neurons with the FDA-approved NMDAR antagonist memantine (50 µM), consistent with clinical reports of NMDAR receptor antagonists being used to treat refractory epilepsy in patients with *GRIN* mutations (Pierson et al., 2014; Li et al., 2016; Amador et al., 2020). Given that ketamine, another FDA-approved NMDAR antagonist, is currently widely used to treat status epilepticus (Ilvento et al., 2015; Zeiler, 2015; Pribish et al., 2020), and has been used to treat refractory epilepsy in patient with a *GRIN2D* mutation (Li et al., 2016), we evaluated its neuroprotective profile in GluN2A-P552R mediated neurotoxicity.

**FIGURE 1:**
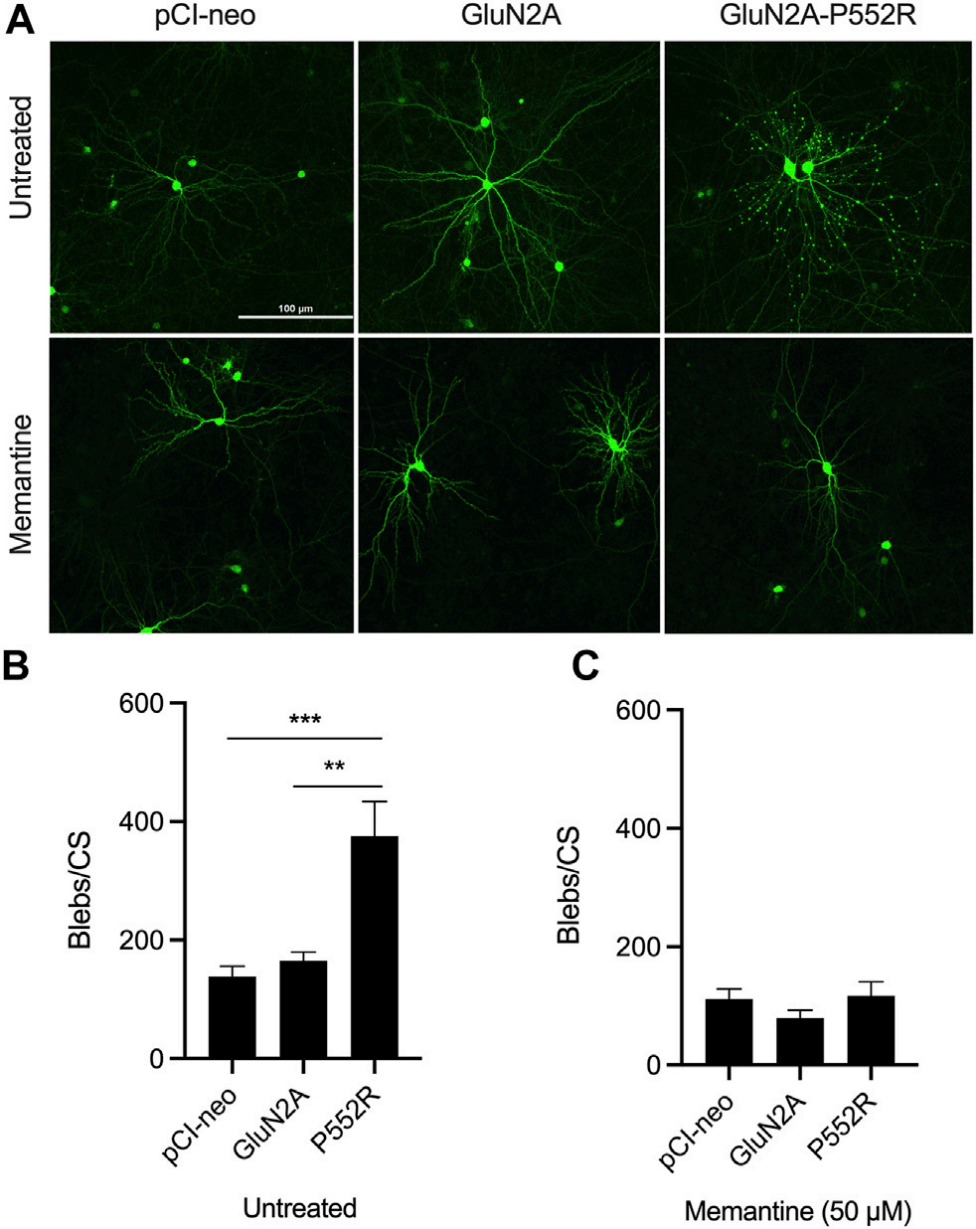
GluN2A-P552R expression exerts dendrotoxicity in rat primary cortical cultures which can be rescued by treatment with memantine. Representative images of cortical neurons transfected with GFP and either pCI-neo (empty vector), GluN2A, or GluN2A-P552R. Top row shows untreated neurons and bottom row shows neurons co-treated with 50 µM memantine **(A)**. Quantification of dendritic blebs per coverslip (blebs/CS) revealed significantly more blebs in GluN2A-P552R transfected neurons as compared to those transfected with pCI-neo or GluN2A **(B)**. This difference is abolished by treatment with 50 µM memantine **(C)**. Data are expressed as mean ± SEM from three independent experiments with three coverslips per transfection condition in each experiment (**p < 0.01, ***p < 0.001, one-way ANOVA/Tukey post-hoc, n = 9).

**FIGURE 2:**
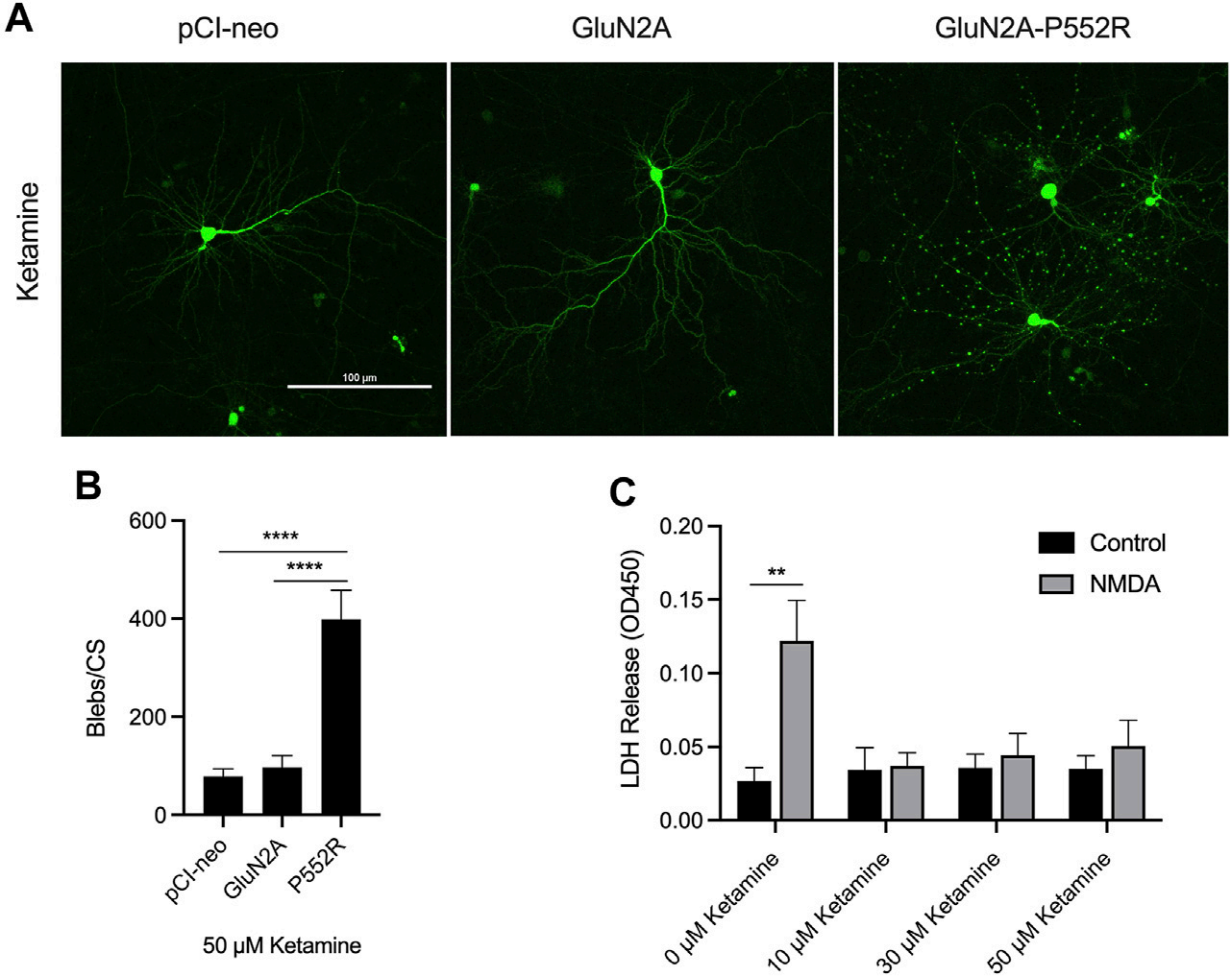
Ketamine treatment does not rescue GluN2A-P552R-mediated dendritic blebbing. Representative images of cortical neurons transfected with GFP and either pCI-neo, GluN2A, or GluN2A-P552R and co-treated with 50 µM ketamine show that GluN2A-P552R transfected neurons exhibit pronounced blebbing that is not rescued by the open-channel blocker **(A)**. Quantification of dendritic blebbing confirmed significantly more blebs in the GluN2A-P552R group **(B)** (****p < 0.0001, one-way ANOVA/Tukey post-hoc, n = 9). LDH assays of untransfected primary cortical neurons treated with 10 µM glycine (control) or 10 µM glycine + 30 µM NMDA (NMDA) confirmed that ketamine is protective against excitotoxic injury **(C)** (**p < 0.01, two-way ANOVA/Sidak post-hoc, n = 3). Therefore, the failure of ketamine to rescue GluN2A-P552R dendrotoxicity is not due to inefficacy of the drug in our culture system. Data are expressed as mean ± SEM from three independent experiments. Three transfections per condition were analyzed from each independent imaging experiment.

We first confirmed our previous findings from the initial characterization of the GluN2A-P552R mutation (Ogden et al., 2017). As noted earlier, cortical neurons transfected with GluN2A-P552R displayed pronounced dendritic blebbing (Ogden et al., 2017) (Figures 1A,B). This effect was not observed in neurons transfected with the empty vector (pCI-neo) or, importantly, the wild-type subunit (GluN2A). GluN2A-P552R-mediated dendrotoxicity was attenuated by treatment with 50 µM memantine (One-way ANOVA, *p* = 0.3025, n = 9) (Figures 1A,C), as previously shown, confirming that FDA-approved NMDAR antagonists may be a viable treatment strategy for individual(s) with this mutation. Surprisingly, although ketamine and memantine share the same mechanism of action, similar pharmacodynamic profiles, and overlapping binding sites within NMDA receptors (Song et al., 2018; Zhang et al., 2021; Emnett et al., 2013), we found that ketamine treatment did not rescue GluN2A-P552R mediated dendritic blebbing (One-way ANOVA, *p* < 0.0001; Multiple comparisons, pCI-neo vs. GluN2A, *p* = 0.9407, pCI-neo vs. GluN2A-P552R, *p* < 0.0001, GluN2A vs. GluN2A-P552R, *p* < 0.0001, n = 9) (Figures 2A,B). Indeed, an unpaired *t*-test revealed that there was no difference in the number of dendritic blebs per coverslip between untreated neurons and those treated with 50 µM ketamine (*p* = 0.7819, n = 9). Although there are some reports of ketamine induced neurotoxicity in neurons exposed to the drug for prolonged periods (Liu et al., 2013; Wang et al., 2017), empty vector transfected neurons and GluN2A transfected neurons, which were also treated with ketamine in parallel with GluN2A-P552R transfected neurons, did not display any dendrotoxicity, suggesting that the dendritic blebbing observed in GluN2A-P552R group was not a result of the ketamine treatment itself. It is noteworthy that ketamine’s ability to inhibit GluN1/GluN2A-P552R-mediated ionic currents does not significantly differ from its ability to block GluN1/GluN2A wild type channels. Ketamine’s maximum blocking efficacy for GluN1/GluN2A-P552R channels is 96% as compared to 98% for wild-type channels. Furthermore, the GluN2A-P552R mutation enhances the potency of ketamine (IC_50_ =1.3 µM for GluN2A-P552R; IC_50_ = 4.7 µM for wild type GluN2A), without significantly affecting memantine’s blocking actions at the receptor (IC_50_ = 3.7 µM for GluN2A-P552R; IC_50_ = 4.8 for wild type GluN2A) (Ogden et al., 2017). As such the lack of ketamine’s ability to protect from the observed dendrotoxicity could not be accounted by an inability to antagonize the mutant channels.

Given these data, we sought to confirm that ketamine was indeed protective against canonical excitotoxic insults in our cell culture system. To this end, we exposed untransfected cortical cultures to either 10 µM glycine (control) or 10 µM glycine and 30 µM NMDA for 30 min and co-treated with either vehicle or ketamine (10 μM, 30 μM, or 50 µM). Cell viability was assessed using LDH assays 24 hs after the exposure. Cells not treated with ketamine displayed a significant loss of viability when exposed to glycine + NMDA as compared to the control, whereas no significant differences were found between the control and glycine + NMDA groups when cells were co-treated with ketamine (Two-way ANOVA, *p* = 0.0158 for interaction; Multiple comparisons, glycine vs glycine + NMDA: 0 µM ketamine, *p* = 0.0021, 10 µM ketamine, *p* = 0.9997, 30 µM ketamine, *p* = 0.9820, 50 µM ketamine, *p* = 0.8645, n = 3) (Figure 2C). Furthermore, comparison of control neurons in these assays confirmed that ketamine treatment is not neurotoxic in our system (One-way ANOVA, *p* > 0.05). These results indicate that ketamine is an effective excitotoxicity neuroprotectant in our preparation.

**FIGURE 3:**
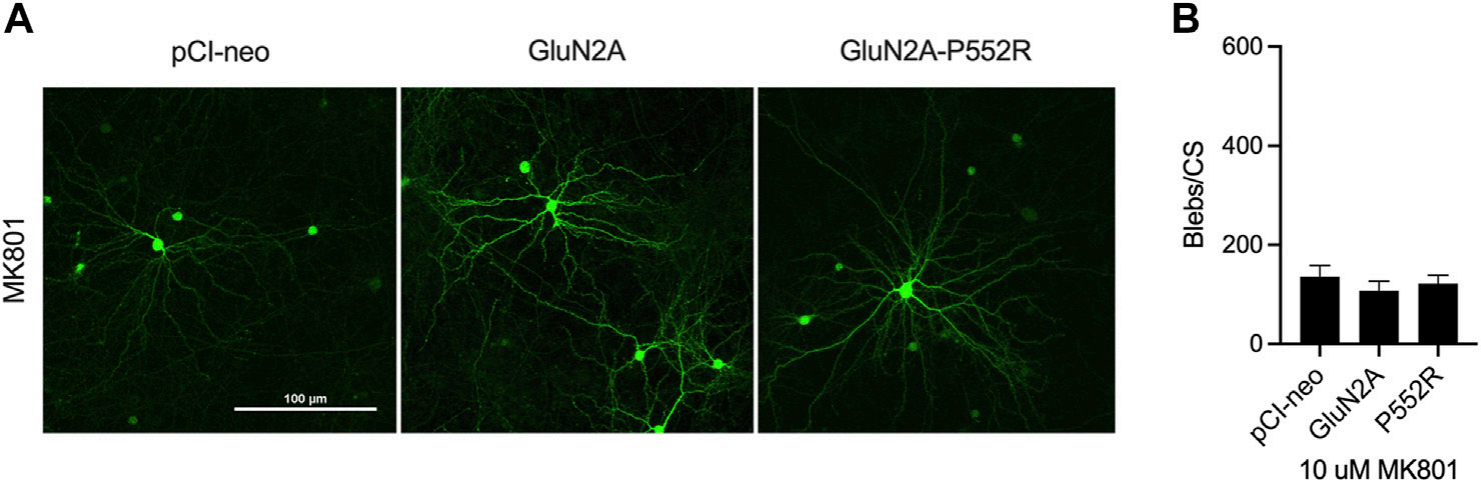
Dendritic blebbing is attenuated by treatment with the channel blocker MK801. Representative images of rat cortical neurons transfected with pCI-neo empty vector, GluN2A-WT, and GluN2A-P552R and co-treated with 10 µM MK801 **(A)**. GluN2A-P552R expressing neurons treated with 10 µM MK801 show a significant reduction in blebbing **(B)** (one-way ANOVA, *p* > 0.05, n = 9). Data are ± SEM from three independent experiments with three coverslips analyzed per experiment.

As the above data indicate that the inability of ketamine to rescue of GluN2A-P552R mediated dendrotoxicity is not due to a loss of potency or innate toxicity, we next aimed to determine whether memantine’s ability to rescue this phenotype was through its classically defined role as an NMDA receptor channel antagonist or through an undefined, alternative mechanism. To this end, we exposed GluN2A-P55R expressing neurons to the open-channel blocker MK-801, which binds to the same pocket of the NMDAR as memantine and ketamine (Song et al., 2018; Zhang et al., 2021). Treatment with 10 µM MK-801 similarly abolished GluN2A-P552R mediated dendritic blebbing (One-way ANOVA, *p* > 0.05) (Figures 3A,B), strongly suggesting that memantine rescues GluN2A-P552R through its known mechanism of pharmacological action.

Having established the NMDAR blockade is sufficient to rescue GluN2A-P552R mediated dendrotoxicity, we next investigated downstream pathways that may be either activated or inhibited by ketamine, but not by memantine, which could account for the differential responses observed. Activation of MAP kinases (MAPKs) have previously been shown to be part of signaling cascades initiated downstream of NMDAR activation (Kawasaki et al., 1997; Chen et al., 2003), including those implicated in excitotoxicity (Kawasaki et al., 1997; Cao et al., 2004; Choo et al., 2012; Liu et al., 2014; Engin and Engin, 2021). Therefore, we assessed via western blot whether there were differences in kinase phosphorylation in primary cortical neurons treated with either ketamine or memantine. No significant differences were found between memantine and ketamine groups in levels of phosphorylated ERK1/2 or JNK (Paired *t*-test, *p* > 0.05, n = 5–6) (Supplementary Figure S1F and S1G). A small but significant reduction in phosphorylated p38 (p-p38) was found in ketamine treated neurons as compared to those treated with memantine (Paired *t*-test, *p* = 0. 0.0036, n = 5) (Supplementary Figure S1E). However, given that phosphorylation of p38 has been implicated in excitotoxic neuronal apoptosis (Kawasaki et al., 1997; Chen et al., 2003; Liu et al., 2014), it is unlikely that the reduction of p-p38 in the ketamine treated group is the mechanism underlying the persistent dendritic blebbing in ketamine treated GluN2A-P552R expressing neurons. As Src kinase has been shown to upregulate NMDAR activity (Manzerra et al., 2001; Zhao et al., 2015; Scanlon et al., 2017), we also assessed whether memantine and ketamine treatment differentially affected Src phosphorylation. However, no differences were found between groups (Paired *t*-test, *p* > 0.05, n = 5) (Supplementary Figure S1H). Thus, the mechanism underlying the inability of ketamine to rescue GluN2A-P552R-induced dendritic blebbing is yet to be identified. Nonetheless, the data presented thus far strongly suggests that human disease-linked *GRIN* mutations may be differentially responsive to NMDAR antagonists.

**FIGURE 4:**
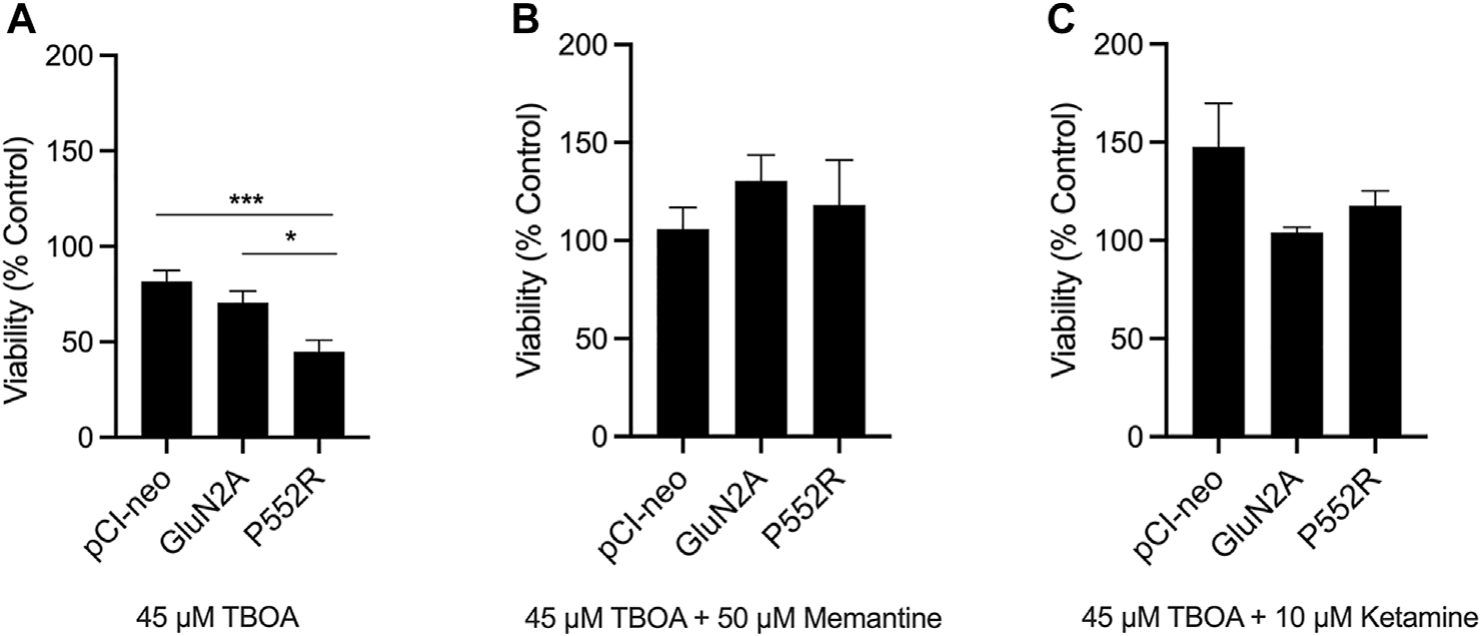
GluN2A-P552R enhances vulnerability to excitotoxic stress which can be rescued by both memantine and ketamine. Luciferase assays were used as a measure of cell viability. Rat cortical neurons were co-transfected with a plasmid expressing firefly luciferase and pCI-neo vector, GluN2A-WT, or GluN2A-P552R. Treatments were applied 24 h after transfection and luciferase assays were performed 48 h following transfection. Neurons expressing GluN2A-P552R showed significantly reduced viability after exposure to 45 µM TBOA as compared to those expressing the empty vector or wild-type receptor **(A)** (*p < 0.05, ***p < 0.001, one-way ANOVA/Tukey post-hoc, n = 8). However, this reduction in viability was rescued by co-treatment with 50 µM memantine **(B)** (one-way ANOVA, *p* > 0.05) or 10 µM ketamine **(C)** (one-way ANOVA, *p* > 0.05). Luciferase values for TBOA treated neurons were normalized to their relevant vehicle-treated vector group. Data represent mean ± SEM from 4-8 independent experiments performed in triplicate or quadruplicate.

Because the GluN2A-P552R mutation results in increased agonist potency and mean channel open time (Ogden et al., 2017), we hypothesized that neurons that express this mutant subunit may show enhanced vulnerability to excitotoxic injury, perhaps due to exaggerated calcium influx upon activation. To test this, we transfected neurons with a plasmid expressing firefly luciferase along with the either the empty vector, GluN2A, or GluN2A-P552R. Co-transfection of the firefly luciferase construct allowed us to compare viability of cells expressing the mutant subunit as compared to wild-type GluN2A or the empty vector (Aras et al., 2008). Twenty-four hs after transfection, neurons were exposed to a mild excitotoxic insult (treatment with a sublethal concentration of the glutamate uptake inhibitor TBOA, 45 µM) for an additional 24 hs. Indeed, following TBOA exposure, we found a significant difference in viability of GluN2A-P552R transfected neurons as compared to those transfected with the empty vector or the wild-type subunit (One-way ANOVA, *p* = 0.001; Multiple Comparisons, pCI-neo vs GluN2A, *p* = 0.3983, pCI-neo vs GluN2A-P552R, *p* = 0.0008, GluN2A vs GluN2A-P552R, *p* = 0.0181). (Figure 4A). Co-treatment with memantine rescued the loss of viability observed in GluN2A-P552R expressing neurons (One-way ANOVA, *p* > 0.05), consistent with its effect on GluN2A-P552R mediated dendrotoxicity (Figure 4B). In contrast to ketamine’s failure to attenuate dendritic blebbing, co-treatment with this channel blocker also completely rescued TBOA-induced cell death in GluN2A-P552R expressing neurons (One-way ANOVA, *p* > 0.05) (Figure 4C). These data strongly suggest that the P552R mutation exerts dendrotoxicity and enhances vulnerability to excitotoxic stress through two separate cell injurious signaling pathways.

## Discussion

The rise of next-generation and whole exome sequencing (WES) has led to the increasing identification of clinically relevant gene variants and molecular diagnoses in patients with neurological diseases. Indeed, in one large observational study, WES was able to provide a molecular diagnosis for approximately 27% of patients with neurological and developmental disorders (Yang et al., 2014). In a smaller cohort of pediatric neurological patients, WES provided a presumptive diagnosis in 41% of patients. Importantly, the results of WES affected the management of all patients with a presumptive diagnosis in this cohort including, but not limited to, the cessation and initiation of medication (Srivastava et al., 2014). Thus, genetic testing has both a high diagnostic yield and significant implications for personalized clinical management for neurological patients.

The clinical utility of genetic sequencing is especially significant for patients with epileptic encephalopathies. It is estimated that the diagnosis yield for these disorders is 15–20% (EpiPM Consortium, 2015). Intriguingly, *GRIN* mutations make up a high proportion of these disease-linked mutations, and *GRIN2A* mutations, specifically, have been identified as key drivers of epilepsy aphasia spectrum disorders (Carvill et al., 2013; Li et al., 2020; Lemke et al., 2013; Lesca et al., 2013). The identification and *in vitro* characterization of human disease-linked *GRIN* mutations has led to the successful implementation of personalized medicine for several pediatric patients. More specifically, patients with *GRIN* mutations and intractable epilepsy have been treated with FDA-approved NMDAR blockers, including ketamine, memantine, and dextromethorphan (Supplementary Table S1), which led to a reduction in seizure burden in the majority of patients treated with these targeted therapies (Pierson et al., 2014; Li et al., 2016; Platzer et al., 2017; Amador et al., 2020; Xu et al., 2021). The results of these *n* of one clinical trials have important clinical implications for patients with *GRIN*-associated epileptic encephalopathies, as refractory epileptic activity in these disorders is thought to contribute to the co-morbid, and often progressive, cognitive impairments (Khan and Al Baradie, 2012; Auvin et al., 2016).

The increasing number of individuals with identified *GRIN* mutations along with the *in vitro* characterization of NMDAR subunit variants has led to an attempt by some to predict clinical phenotypes based upon molecular diagnoses. For example, it has been suggested that *GRIN2A* mutations predominantly lead to an epileptic phenotype while *GRIN2B* variants are more likely to lead to neurodevelopmental disorders (Myers et al., 2019). However, while this may be true in broad strokes, *GRIN2B* has been identified as a causative gene in encephalitic encephalopathies (Epi4K Consortium et al., 2013; Lemke et al., 2014). Moreover, the characterization of *GRIN2B* mutations in two patients with missense mutations at the same amino acid residue revealed a divergence in multiple aspects of both the patient phenotypes and the functional properties of the mutant channels (Kellner et al., 2021), underscoring the complexity of the NMDAR and the need for complete characterizations of *GRIN* variants for targeted therapies. Similarly, characterizing *GRIN* mutations as gain-of-function or loss-of-function based on their location in the protein domain may be overly simplistic and lead to improper pharmacological treatment strategies. While a large cohort analysis of individuals with *GRIN2A* variants identified two phenotypic groups based on whether mutations occurred within the N-terminal or agonist binding domains versus the transmembrane or linker domains, with the former corresponding to loss-of function (LOF) and the latter gain-of-function (GOF) (Strehlow et al., 2019), functional analysis of disease-linked mutations within the pre-M1 linker domain of GluN2A receptors identified both LOF and GOF variants (Ogden et al., 2017). Thus, a full functional characterization of *GRIN* mutations is warranted before the initiation of treatment with channel-blockers.

In this work, we uncover another layer of complexity in the application of precision medicine to *GRIN* mutations: the differential response of a mutant subunit to channel blockers that do not differ in their potency or maximal inhibition of the receptor. Specifically, we found that a missense *GRIN2A* variant resulting in a substitution of arginine for proline at amino acid 552 (P552R) exerts neurotoxicity through two distinct mechanisms—increased sensitivity to excitotoxic insults, which is sensitive to rescue by the channel blockers memantine and ketamine, and pronounced dendrotoxicity that is sensitive to rescue by memantine and MK801, but not ketamine.

Previous characterizations of *GRIN* mutations have found differential responses to channel blockers. For example, treatment with dextromethorphan results in increased inhibition of the NMDA channel as compared to memantine in the NMDA receptor GluN2AN-N615K variant (Marwick et al., 2019). However, memantine binds to the pore by interacting with the amino acids that cluster around the area of this mutation (N612, N613, and N614) (Song et al., 2018), while dextromethorphan is thought to interact with residues in a more extracellular portion of the vestibule (LePage et al., 2005). Thus, increased inhibition of GluN2A-N615K containing receptors by dextromethorphan is consistent with the understanding of its binding site, while differences in binding sites cannot explain the observed differential dendro-protective response of GluN2A-P552R-containing NMDA receptors to memantine and ketamine, as these channel blockers bind to an overlapping site in the pore (Emnett et al., 2013; Zhang et al., 2021). To account for the noted changes, we explored whether memantine and ketamine differentially activate or inhibit kinases downstream of neurotoxic NMDAR signaling. However, these experiments did not reveal significant differences between treatment groups. Given that memantine has been found to stabilize GluN1/GluN2A receptor desensitization in a calcium-dependent manner (Gao et al., 2017), one possible explanation for the discrepancy between memantine and ketamine’s ability to rescue GluN2A-P552R mediated dendrotoxicity is that the mutation enhances calcium influx through NMDARs thereby enhancing memantine’s potency. However, preliminary experiments showed that the IC50 of memantine in the presence of high and low intracellular calcium did not differ between wild-type and mutant receptors, suggesting this phenomenon does not account for our results (M. Phillips and J. Johnson, personal communication). Thus, the mechanism underlying the differential response of GluN2A-P552R containing NMDARs to memantine and ketamine remains to be elucidated. Nonetheless, our findings are significant in the context of personalized medicine for patients with neurological disorders attributable to *GRIN* mutations as they suggest that in addition to a functional characterization of the mutation, *in vitro* drug screening and investigation of rescue pharmacology may be necessary to identify the most appropriate therapeutic strategies to follow.

## Data Availability

The original contributions presented in the study are included in the article/Supplementary Material, further inquiries can be directed to the corresponding author.
