# Supplementary material for: Imprecision in Precision Medicine: Differential Response of a Disease-Linked GluN2A Mutant to NMDA Channel Blockers
Source: Front Pharmacol. 2021 Oct 28;12:773455. doi: 10.3389/fphar.2021.773455 (PMC8581401; doi:10.3389/fphar.2021.773455)
Supplement: Supplementary file 1 [file Table1.docx]

Supplementary Material

**Supplemental Table 1.** Summary of patients with *GRIN* mutations treated with NMDAR antagonists.

| **Gene** | **Mutation** | **Patient Phenotype** | **Treatment** | **Clinical Response** | **Citation** |
| --- | --- | --- | --- | --- | --- |
| GRIN1 | p.M64I | Early onset epileptic encephalopathy, severe developmental delay | Memantine (added as an adjunct therapy) | Decrease in frequency and severity of seizures | Xu et al. (2021) |
| GRIN2D | p.V667I | Intractable epilepsy, global developmental delay, static encephalopathy | Memantine | Decreased seizure burden and improved developmental status | Li et al. (2016) |
| GRIN2D | p.Vl667I | Epilepsy resistant to standard anti-epileptic therapy, global developmental delay, microcephaly | Memantine, Ketamine and magnesium | No clinical improvement following treatment with memantine, dramatic reduction in seizures following ketamine + magnesium treatment | Li et al. (2016) |
| GRIN2A | p.L812M | Epileptic encephalopathy, intractable seizures, profound cognitive impairment, absent motor development | Memantine (added as an adjunct therapy) | Decrease in seizure activity, no cognitive improvement | Pierson et al (2014) |
| GRIN2A | p.S644G | Epileptic encephalopathy, intractable epilepsy, global developmental delay | Memantine followed by addition of dextromethorphan | Significant decrease in seizure burden, no cognitive/developmental improvement | Amador et al. (2020) |
| GRIN2B | p.N615I | Recurrent epileptic spasms and tonic clonic seizures, severe intellectual disability | Memantine | No improvement in seizure activity but subjective improvement in awareness | Platzer et al. (2017) |
| GRIN2B | p.V618G | Severe intellectual disability, seizure-free with standard anti-epileptic therapy | Memantine (added as an adjunct therapy) | Subjective improvement in awareness and decreased restlessness | Platzer et al. (2017) |
| GRIN2B | p.G611V | Severe intellectual disability, generalized seizures | Memantine | No change in seizure frequency but subjective improvement in walking, awareness, social interactions, and sleep pattern | Platzer et al. (2017) |
| GRIN2B | p.M818T | Developmental delay, focal and generalized seizures | Memantine (added as adjunct) | No change in seizure frequency but subjective improvement in awareness | Platzer et al. (2017) |
