# Supplementary material for: Imprecision in Precision Medicine: Differential Response of a Disease-Linked GluN2A Mutant to NMDA Channel Blockers
Source: Front Pharmacol. 2021 Oct 28;12:773455. doi: 10.3389/fphar.2021.773455 (PMC8581401; doi:10.3389/fphar.2021.773455)
Supplement: Supplementary file 2 [file DataSheet1.docx]

Supplementary Material

# Supplementary Figures

**Supplementary Figure 1.** Treatment with memantine and ketamine does not cause differential activation or inhibition of kinases in primary cortical neurons. Western blots samples from cortical neurons treated for 45 minutes with either vehicle, 50 µM memantine or 10 µM ketamine **(A-D)**. Quantification of memantine and ketamine treated neurons, calculated as the ratio of phosphorylated p38/p38 **(E)**, phosphorylated pERK 1/2/ERK 1/2 **(F)**, pJNK/JNK **(G)**, and pSrc/Src (after normalization to β-actin) **(H)**, normalized to ratios in vehicle treated neurons. Ketamine treatment significantly reduced p38 phosphorylation as compared to memantine treatment **(E)** (**p<0.01, paired t-test, p=0.0036), however, no other differences between treatment groups were found **(F-H)** (paired t-test, p>0.05). Data represent mean ± SEM from 5-6 experiments with at least 3 separate culture dates.

**Supplementary Figure 2**. Full Western blot of panel A from Supplementary figure 1. Samples from cortical neurons treated for 45 minutes with either vehicle, 50 µM memantine or 10 µM ketamine and probed for phosphorylated p38 **(A)** or p38 **(B)**.

**Supplementary Figure 3**. Full Western blot of panel B from Supplementary figure 1. Samples from cortical neurons treated for 45 minutes with either vehicle, 50 µM memantine or 10 µM ketamine and probed for phosphorylated ERK 1/2 **(A)** or ERK 1/2 **(B)**. Samples from 3 experiments shown from 2 separate culture dates.

.

**Supplementary Figure 4**. Full Western blot of panel B from Supplementary figure 1. Samples from cortical neurons treated for 45 minutes with either vehicle, 50 µM memantine or 10 µM ketamine and probed for phosphorylated JNK **(A)** or JNK **(B)**. Samples from 3 experiments shown from 2 separate culture dates.

**Supplementary Figure 5**. Full Western blot of panel A from Supplementary figure 1. Samples from cortical neurons treated for 45 minutes with either vehicle, 50 µM memantine or 10 µM ketamine and probed for phosphorylated Src **(A)** and β-actin **(B)** or Src **(C)** and β-actin **(D)**. Samples from 2 experiments shown.
